# Prognostic Study of Colorectal Cancer: Differences between Screen-Detected and Symptom-Diagnosed Patients

**DOI:** 10.3390/cancers16193363

**Published:** 2024-09-30

**Authors:** Sergio A. Novotny, Vidina A. Rodrigo Amador, Jordi Seguí Orejuela, Adriana López-Pineda, José A. Quesada, Avelino Pereira-Expósito, Concepción Carratalá-Munuera, Juan Hernandis Villalba, Vicente F. Gil-Guillén

**Affiliations:** 1 Department of General and Digestive Surgery, Elda General University Hospital, Carretera de Sax s/n, 03660 Elda and San Juan de Alicane, Spain; novotny_ser@gva.es (S.A.N.);; 2 Department of Clinical Medicine, Miguel Hernández University of Elche, Crta. Nacional 332 s/n, 03550 San Juan de Alicante, Spainvgil@umh.es (V.F.G.-G.); 3 Research Unit, Elda General University Hospital, Carretera de Sax s/n, 03660 Elda and San Juan de Alicane, Spain

**Keywords:** colorectal cancer, early detection of cancer, mortality, diagnostic screening programmes

## Abstract

**Simple Summary:**

Colorectal cancer (CRC) is a leading cause of death in Spain, but early detection through screening can significantly reduce the risk of death. However, many people in Spain do not participate in these screening programs, like the faecal occult blood test and colonoscopy. To better understand the benefits of screening, we conducted a study comparing patients diagnosed through screening with those diagnosed after developing symptoms. Our study included 315 people diagnosed with CRC at a public hospital in Elda, Spain, from 2014 to 2018, with follow-up until 2023 or death. We found that patients diagnosed through screening were more likely to have early-stage cancer and a family history of CRC, while those diagnosed after symptoms appeared were more likely to have advanced cancer and other chronic diseases. People diagnosed based on symptoms had a higher risk of death from CRC and other causes. Our findings show that CRC screening leads to earlier diagnosis, improving survival rates. These results support the need for public health policies that encourage widespread participation in CRC screening programs to save lives.

**Abstract:**

**Background and objective:** Colorectal cancer (CRC) is the leading cause of mortality in Spain, with screening programs, such as the faecal occult blood test and colonoscopy, having shown effectiveness in reducing CRC incidence and mortality. Despite these advancements, CRC screening uptake remains low in Spain, highlighting the need for studies comparing outcomes between screening-diagnosed and symptom-diagnosed patients to better understand the impact on overall survival and to quantify the clinical benefit in prognosis at diagnosis and at the end of follow-up. **Methods:** We conducted a retrospective cohort study with the following objectives: to compare stage at diagnosis, all-cause mortality, and disease-specific mortality among people diagnosed with CRC based on screening and based on symptoms; to identify the risk factors associated with mortality in this population; and to evaluate the effectiveness of screening on survival and early detection. Our study included people diagnosed with CRC in the public hospital of Elda (Spain) from 2014 to 2018; follow-up was until 2023 or death. Our primary outcome was all-cause mortality, which we analysed using Kaplan–Meier curves. We also investigated CRC-specific mortality and other-cause mortality. **Results**: Our sample included 315 people (186 with symptom-based diagnoses, 129 with screening-based diagnoses). The mean length of follow-up was 62.8 months. The screening group had a higher prevalence of a family history of CRC (*p* = 0.008), a distal tumour location (*p* = 0.002), and a cancer stage of 0 or I (*p* < 0.001). The symptoms group had a higher prevalence of a proximal CRC (*p* = 0.002), other chronic diseases (*p* < 0.001), and stages II, III, and IV (*p* < 0.001). Two variables were associated with mortality: stage IV at diagnosis and previous cancers. People with a symptom-based diagnosis had a higher prevalence of stage IV at diagnosis and a higher cumulative incidence of CRC mortality and all-cause mortality at the end of follow-up (*p* < 0.05). The Kaplan–Meier curves also showed a higher rate of all-cause mortality in the symptoms group throughout the follow-up. **Conclusion**: CRC screening enables an earlier diagnosis and improves survival. These findings support public health policies that promote accessible and effective screening.

## 1. Introduction

Cancer is the leading cause of death in people aged under 65 years in Europe [1]. Colorectal cancer (CRC) is the most prevalent type in the Western countries and the leading cause of death in Spain, when both sexes are considered [2,3]. Its pathogenesis and risk factors are well established [4]. The individual risk depends on age, sex, genetic factors, a family history of CRC, smoking, alcohol consumption, physical activity, and the gut microbiota [5]. Some 85% of CRC cases are sporadic and have adenomatous polyps (60% to 70%) or serrated lesions (15% to 30%) as precursor lesions. Because these lesions take 10 to 15 years to develop into cancer, systematic population screening is recommended to ensure early detection [6,7]. However, many cases of CRC are diagnosed after the appearance of signs and symptoms such as bowel transit disturbances, anaemia, rectorrhagia, obstruction, perforation, palpable abdominal or rectal masses, and weight loss [7].

In 2003, the European Council recommended the implementation of CRC screening based on the faecal occult blood test (FOBT) for men and women aged 50 to 74 years [8]. The immunological FOBT (iFOBT) is more effective for identifying CRC and advanced adenomas than the guaiac FOBT (gFOBT) [9]. In addition, cohort studies have shown that colonoscopy achieves a significant reduction in CRC incidence and mortality [10]. The 2021 U.S. Preventive Services Task Force (USPSTF) recommendations call for any screening test (gFOBT or iFOBT every year, computed tomography (CT) colonography every five years, flexible sigmoidoscopy every five years, flexible sigmoidoscopy every ten years plus annual iFOBT, or colonoscopy every 10 years) to diagnose CRC in people aged 50 to 75 years (highest evidence recommendation), with varying degrees of recommendation for other age groups [10]. The U.S. Multi-Society Task Force on Colorectal Cancer suggests starting screening at age 45 years in medium-risk individuals, although there is limited clinical data on the added benefit of screening before age 50 years [11]. Currently, CRC screening is accepted in medium-risk individuals aged 50 to 69 years, with strong evidence for the FOBT, sigmoidoscopy, and colonoscopy [7,12,13].

Despite these recommendations, seven European Union Member States currently have no CRC screening programme in place [14]. European guidelines recommend participation rates above 65% to 70% to achieve a cost-effective reduction in CRC mortality [15], but current rates are approximately 40% in countries such as Australia [16] or Spain [17] (with considerable variability between different Autonomous Communities). The implementation of CRC screening programs in Europe has shown significant variability in participation rates and the stage at which cancers are detected, highlighting the ongoing challenges in achieving effective screening coverage [18].

Participation in CRC screening is significantly affected by socioeconomic factors. People with a lower education level or income and those in rural areas are less likely to participate. The European Health Interview Survey (2018–2020) [19] showed that a younger age, not living with a partner, and limited healthcare access also correlate with lower screening rates. For instance, countries with comprehensive screening programs, like Denmark, have higher participation, while those with partial or no programs, such as Bulgaria, see lower rates. This highlights the need to improve awareness and accessibility, particularly in underserved areas.

A CRC screening programme was incorporated into the common portfolio of services of the Spanish National Health System in 2014 [20]. This screening is conducted biennially, according to the standards approved by the Spanish Ministry of Health, and targets men and women aged 50 to 69 years who are medium-risk and uses the iFOBT. To improve participation and thus oncology outcomes, these programmes must meet quality criteria related to awareness raising, the repetition of messages, and sensitivity to tone and style [21].

CRC survival has increased in recent years [13]. Some studies have found that screening leads to earlier CRC detection and reduced CRC mortality. However, there is scarce evidence on all-cause mortality from population-based studies. It is important to establish whether people diagnosed with CRC by screening live longer than those diagnosed by clinical presentation, and to estimate the magnitude of the difference. This information could be useful for motivating the population to get tested [12,13,22,23]. Brenner et al. [24] found that people with CRC that was detected by screening had a better prognosis than those diagnosed by symptoms, even after controlling for the CRC stage. This suggests that screening not only helps detect cancer at earlier stages but may also be associated with tumour or host characteristics that result in better survival outcomes. Therefore, we aimed to evaluate the impact of screening in the municipality of Elda (Spain) since the implementation of CRC population screening in 2014. The objectives of this study were to compare stage at diagnosis, all-cause mortality, and CRC mortality among people with a CRC diagnosis based on screening versus on symptoms; to identify the risk factors associated with all-cause mortality in this population; to estimate the impact of CRC screening-based versus symptom-based diagnoses on the prevalence of advanced stages at diagnosis and on the cumulative incidence of mortality at the end of follow-up; and to assess the effectiveness of screening in terms of survival and early disease detection.

## 2. Material and Methods

We conducted a retrospective cohort study from December 2019 to February 2023, in the municipality of Elda (Alicante, Spain). The Ethics Committee of Elda General University Hospital approved the study protocol (Date: 29 October 2019), which complies with the Declaration of Helsinki.

Using the hospital discharge programme, we selected people aged 50 to 69 years who were diagnosed with any stage of CRC (International Classification of Diseases (ICD)-9 code 153.0 to 154.1: colon cancer, cecum cancer, ascending colon cancer, splenic angle cancer, descending colon cancer, sigmoid cancer, rectal cancer) in a public hospital in Elda between 2014 and 2018, inclusively. The diagnosis could be based on screening or symptoms. We excluded people who chose to be treated in a private centre after the initial diagnosis, due to the lack of follow-up data. Other exclusion criteria were a previous diagnosis of any type of hereditary polyposis, a diagnosis of hereditary non-polyposis CRC, and a history of inflammatory bowel disease, since they are subjected to specific endoscopic monitoring different from the rest of the population due to being a high-risk group.

In non-urgent cases, diagnoses were confirmed by endoscopy and pathology, while in urgent cases requiring surgery, diagnoses were confirmed by CT and pathology. The date of diagnosis was considered the date of endoscopy/CT scan when these test results were conclusive, or the date of pathological confirmation in all other cases.

We divided participants into two groups: those who underwent diagnostic testing after iFOBT screening (screening group) and those who underwent diagnostic testing due to symptoms (symptoms group). We performed a retrospective follow-up of both cohorts from the screening/presentation of CRC symptoms until the time of death or the study’s completion (3 February 2023).

Our primary outcome was all-cause mortality. We also assessed CRC mortality and mortality from other causes (any death not related to CRC) separately. Our independent variables were age; sex; the route of CRC diagnosis (screening or symptoms); the stage with 8th edition TMN classification [25] (stages 0 to IV) at the end of initial CRC staging though a pathological evaluation (pTNM); a family history of CRC (yes/no); comorbidities, including diabetes, hypertension, depression, other chronic diseases (yes/no); tumour location (proximal, distal, rectal); cardiovascular events (yes/no); neurological and cerebrovascular events (yes/no); a previous diagnosis of another tumour (yes/no); and treatment with anticoagulants (yes/no). We selected these variables based on previous studies and the possibility of confounding the dependent variable. The staging was assigned by the treating oncologist for each patient and was subsequently reviewed by three independent evaluators who verified the extracted data; two were surgeons with more than 10 years of experience in their speciality, and the other was a final year surgical resident.

We extracted data from the participants’ electronic medical records to an Excel data collection form. Using participants’ unique medical record numbers, we confirmed that no participants had been included in both groups. For all the participants who died before the study’s completion, we collected the cause of death from their electronic medical record. The physician who recorded the time of death was either an oncologist, internist, general surgeon, primary care physician, or a home hospital service physician (in one case). The three data evaluators for the study verified the extracted data; two were surgeons with more than 10 years of experience in their speciality, and the other was a final year surgical resident. We anonymised all the patient data, assigning an ID number to each case.

### Statistical Analysis

We performed a descriptive analysis, calculating absolute and relative frequencies for qualitative variables and the mean with standard deviation (SD) or the median with interquartile range (IQR) for quantitative variables. We used contingency tables to analyse the factors associated with all-cause mortality, applying the Chi-square test for qualitative variables and the Student’s *t*-test or the Mann–Whitney U test for quantitative variables. To estimate the magnitudes of association between the group and the symptoms and the explanatory variables, multivariate binary logistic models were adjusted, estimating the Odds Ratio (OR) and their 95% confidence intervals (95% CI). The goodness of fit of the model was checked using the Likelihood Ratio Test (LRT) and the area under the ROC curve was estimated with its 95% CI.

We compared the prognosis of the screening group and the symptoms group at two time points: at diagnosis (a cross-sectional analysis) and at the end of the follow-up. We compared the prevalence of different disease stages in the two groups by calculating the prevalence ratio (PR), attributable risk (AR), and exposure-attributable fraction (EAR). Patients diagnosed by symptoms were considered exposed and those diagnosed by screening not exposed. We compared the prevalences of stage IV versus stage 0-III patients in each group. At the study’s completion, we assessed all-cause mortality, CRC mortality, and other-cause mortality by cumulative incidence, calculating the relative risk (RR), AR, and AEF. To evaluate the precision of the results, we determined 95% confidence intervals (CIs) for all the measures of frequency, association, and impact, both at diagnosis and at the study’s completion.

Cumulative mortality incidences were estimated according to the explanatory variables using contingency tables and the Chi-square test. We created Kaplan–Meier curves for all-cause mortality, applying the log rank test for the factors of interest. To estimate the magnitudes of associations with all-cause mortality, we fitted Cox survival models, using a stepwise variable selection procedure based on the AIC (Akaike Information Criterion). We calculated hazard ratios (HRs) with their 95% Cis, as well as goodness-of-fit indicators and predictive indicators such as the C-index. The analyses were performed using SPSS v.28 and R v.4.2.2.

## 3. Results

We initially selected 317 eligible patients, but two of them chose to be treated in private hospitals and were excluded. Of the 315 study participants, 186 were diagnosed by symptoms and 129 by screening. The mean follow-up time was 68.2 months (SD 29.0).

Table 1 shows the descriptive characteristics of all the participants and of each study group, together with their bivariate analysis. More than two-thirds of patients (68.3%; n = 215) were men, 10.5% (n = 33) had a family history of CRC, 15.9% (n = 50) had diabetes, 38.1% (n = 120) had hypertension, 74% (n = 233) had other chronic diseases, and 14.6% (n = 46) had a previous cancer diagnosis. The most frequent tumour location was distal (36.8%, n = 116), and the most prevalent stages were stage III (32.7%, n = 103), stage I (22.2%, n = 70), and stage II (21.0%, n = 66).

The results of the bivariate analysis between the study groups show five statistically significant variables. The screening group had a higher prevalence of family history of CRC (*p* = 0.008), distal tumour location (*p* = 0.002), and stages 0 and I CRC (*p* < 0.001). The symptoms group had a higher prevalence of proximal location (*p* = 0.002), other chronic diseases (*p* < 0.001), and stages II, III, and IV CRC (*p* < 0.001). We found no significant differences between the groups in terms of sex, age, diabetes, hypertension, depression, cardiovascular event, stroke or neurological event, previous cancer diagnosis and anticoagulant treatment. (Table 1).

Table 2 shows the multivariable logistic model of the comparison between CRC diagnoses by symptoms and by screening. Five variables were statistically significant. The symptoms group had a higher prevalence of stage II CRC (OR 4.327, *p* = 0.0063), stage III CRC (OR 3.661, *p* = 0.0113) stage IV CRC (OR 5.732, *p* = 0.0023), diabetes (OR 2.308, *p* = 0.0354), proximal involvement (OR 2.444, *p* = 0.0096), and other chronic diseases (OR = 1.999, *p* = 0.0208), and a lower prevalence of a family history of CRC (OR 0.291, *p* = 0.0043). The area under the receiver operating characteristic curve (AUC) was 0.774 (95% CI 0.723 to 0.825, *p* < 0.001).

All-cause mortality at the end of follow-up was 24.1% in the whole study sample. More people in the symptoms group died compared with those in the screening group (28.5% versus 17.8%, *p* = 0.030). Of all deaths, the proportion attributable to CRC was 71.1% in the whole study sample, 73.6% in the symptoms group, and 65.22% in the screening group. Table 3 shows the frequency measures and the calculations of association and impact for tumour stage at diagnosis (IV versus 0-III) and mortality at the end of the follow-up (all-cause mortality, CRC mortality, and other-cause mortality). In the symptoms group, there was a significantly higher prevalence of stage IV cancer at the time of diagnosis, and higher CRC mortality and all-cause mortality at the end of the follow-up (*p* < 0.05).

**Figure 1:**
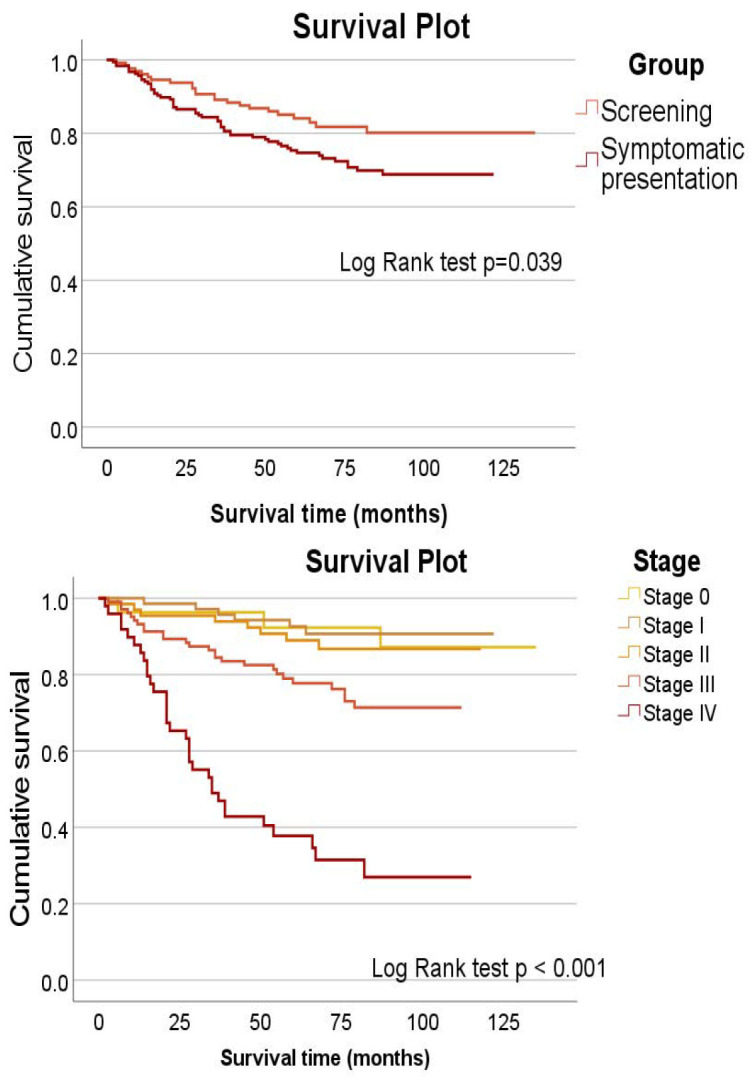
Cumulative survival by type of diagnosis (**top**) and by stage (**bottom**).

Figure 1 top shows the survival curves for both study groups, and Figure 1 bottom shows survival by stage. The participants in the screening group lived longer (*p* = 0.039), and the participants diagnosed with stage III and, especially, stage IV at the end of diagnostic testing had a shorter survival (*p* < 0.001).

Table 4 presents the bivariate analysis of mortality with the explanatory variables of the study. Four variables were statistically significant: total mortality was higher in the symptoms group (*p* = 0.030), in participants with other chronic diseases (*p* < 0.001), in older participants (*p* = 0.027), and in those with stages III and IV CRC (*p* < 0.001).

Table 5 presents the Cox multivariable survival analysis for total mortality. Only two variables were associated with mortality in the staging model: a previous cancer diagnosis (HR 2.57, *p* = 0.002) and stage IV CRC (HR 12.35; *p* < 0.001). The C-index of the multivariable analysis was 0.760 (95% CI 0.707 to 0.813; *p* < 0.001).

## 4. Discussion

We conducted this study to assess the impact of the CRC population-screening programme implemented in the public hospital of Elda in 2014. We evaluated the protective effect and the clinical relevance of screening by comparing a cohort of people diagnosed based on screening against a cohort of people who received diagnostic testing after experiencing CRC symptoms. The two groups were similar in terms of age and sex distribution, had the same diagnostic and therapeutic protocols, and were evaluated over the same follow-up period. The diagnosis of CRC by symptoms, compared with a diagnosis by screening, showed a significant association with a higher percentage of stage IV cancer at diagnosis, higher all-cause mortality, and higher CRC mortality. We found that tumour location was more frequently distal in people diagnosed by screening and proximal in those diagnosed by symptoms. In addition, people diagnosed by symptoms had a higher prevalence of other chronic diseases, including diabetes, and a lower prevalence of a family history of CRC. Although our findings align with the existing literature on the association between stage IV at diagnosis and mortality, as well as the impact of previous cancers, the value of our study lies in its focus on the implementation and real-world effectiveness of a colorectal cancer screening program. By comparing prognoses between screening-diagnosed and symptom-diagnosed patients, we offer important insights into the public health benefits of such screening initiatives.

When we analysed the impact of CRC diagnosis, the screening group had a 53% lower prevalence of stage IV cancer at diagnosis and a 38% lower prevalence of all-cause mortality at the end of follow-up, compared with the symptoms group. The main prognostic marker in CRC is the TNM stage at the time of diagnosis. People diagnosed with stages 0 to III have a higher probability of curative surgical resection than those diagnosed with stage IV. The overall rate of curative resection for CRC ranges from 50% to 60%, and in specialised centres it is almost 80%. More than 40% of stage II or III patients will have a recurrence after such curative treatment, and 80% of recurrences occur within the first two years after surgery [26,27]. The primary prognostic marker for colorectal cancer (CRC) is the TNM stage at the time of diagnosis, with higher stages associated with a worse prognosis. However, the prognosis for CRC has improved because intensive follow-up strategies using colonoscopy and carcinoembryonic antigen (CEA) monitoring reduce overall mortality and increase the chances of performing curative surgery for recurrences [28,29].

In this study, around one in four participants died over a mean follow-up of 5.7 years. For every 10 deaths, seven were due to CRC and three to other causes. The multivariable analysis showed higher all-cause mortality at the end of the follow-up in the symptoms group compared with the screening group. In addition, the screening group had earlier CRC stages, which explains the lower CRC mortality. Brenner et al. [24] also investigated all-cause mortality in people diagnosed by screening compared with those diagnosed by symptoms and found similar results, with overall mortality at 24% and 78% of deaths associated with CRC over 4.8 years of follow-up. They also reported a reduction in all-cause mortality when comparing the TSOH group with the symptomatic group, mainly due to the greater reduction in CRC mortality. There was no statistical significance between the groups in the comparison of mortality from other causes. The Cienfuegos study [3], which compared people diagnosed by screening (n = 250) and by symptoms (n = 1330), also reported a longer survival in those diagnosed by screening. Lastly, Pande et al. [30] reported significantly higher overall survival in patients diagnosed through screening and a trend similar to that of Brenner and the present study when comparing by stages.

Another similar study [28], with 194 participants in the FOBT screening group and 352 participants in the symptoms group, reported a 39% reduction in mortality in the screening group over 3.3 years of follow-up. Inada et al. [29] followed 145 screening-diagnosed and 123 symptom-diagnosed patients for 2.7 years and found a better short-term postoperative outcome and a better long-term oncological outcome in the screening group. However, they found no difference in mortality between the two groups because the study was restricted to postoperative mortality in the first 30 days. Other population-based studies have also reported higher survival rates in screening groups compared with symptoms groups at lower stages of CRC [3,31,32], and Gill et al. [33] found a higher five-year survival rate in screened patients. Further investigation is needed to optimize CRC screening strategies, particularly in understanding the factors that lead to lower screening uptake in certain populations. Addressing these barriers could significantly improve the effectiveness of CRC screening programs and ensure that more individuals benefit from early detection.

One possible explanation for why people diagnosed by screening have earlier-stage tumours and lower CRC mortality is that tumours diagnosed by symptoms, in addition to being detected later than those diagnosed by screening, may have a more aggressive biological phenotype and faster growth [3,24,31,32]. This could be related to an anticipation bias and a duration bias. An anticipation bias occurs as a result of early diagnosis, so that survival is artificially prolonged. Theoretically, if both groups had been diagnosed for symptoms, they would have the same survival time [34,35,36]. A duration bias occurs when slower-growing tumours, which have a longer latent or presymptomatic phase, are diagnosed in screening programmes. These tumours are more likely to be diagnosed by screening, meaning survival in this group is also over-estimated [34,35,36]. Finally, these patients will have a more exhaustive follow-up, leading to the earlier diagnosis of other pathologies. It is necessary to study this cohort for a longer follow-up period to assess whether there are differences in other causes of mortality. Moreover, the role of comorbidities in influencing CRC prognosis warrants further investigation, as our findings suggest that conditions like diabetes may affect tumour stage at diagnosis and overall survival outcomes.

The limitations of this study include the secondary data source (medical records), although three independent evaluators validated all the information. As the data were retrospective, there could be a potential measurement bias; however, this risk should be minimal because cancer deaths are usually documented comprehensively. Additionally, the patients who chose to be treated in a private centre after the initial diagnosis were excluded from this study due to the lack of follow-up data. This exclusion may introduce a potential selection bias and limit the generalizability of our findings. The eligible age range of patients is justified by objective 10 of the Spanish National Health System Cancer Strategy, which indicates that screening should be performed in people aged 50 to 69 years [5,6]. This explains why our study had a lower mean age than Brenner et al., although the distribution by sex and stage were very similar [24].

## 5. Conclusions

Our results show the following: (a) that people diagnosed by screening have earlier-stage tumours compared with people diagnosed by symptoms, and (b) that people diagnosed at earlier stages live significantly longer. This emphasises the importance of screening for the early detection of CRC. We also found that tumour location is more frequently distal in people diagnosed by screening and proximal in those diagnosed by symptoms, and that symptom-diagnosed patients have a higher prevalence of other chronic diseases. These findings may guide future research on the factors associated with tumour location and the coexistence of other diseases, as well as their impact on prognosis. Our results suggest that a family history of CRC and the presence of comorbidities, such as diabetes, may influence the clinical presentation and the stage of CRC, underscoring the need for personalised management and special considerations in prevention and treatment. The higher rates of all-cause and CRC mortality in the symptom-diagnosed group compared with the screening group strengthens the evidence for the survival benefits of screening, which may encourage public health decision makers to improve the accessibility and the promotion of screening programmes. Further investigation is needed to optimize CRC screening strategies, particularly in understanding the factors that lead to a lower screening uptake in certain populations. It is important to note that these conclusions are specific to the population studied, which included only those patients who met our defined inclusion criteria. In particular, the patients who chose private treatment post-diagnosis were excluded from this study, which may limit the generalizability of our findings. Therefore, these results should be interpreted with caution, and further studies are needed to confirm these findings in broader populations.

## Figures and Tables

**Table 1:** Characteristics of study participants (all participants, those with screening-based diagnoses, and those with symptom-based diagnoses).

	Total Sample		Screening-Based Diagnosis		Symptom-Based Diagnosis		
Variable ^a^	n	%	n	%	n	%	*p* Value
**Sex**							
Man	215	68.3%	91	70.5%	124	66.7%	0.467
Woman	100	31.7%	38	29.5%	62	33.3%	
**Age**, mean (SD)	61.1 (5.9)		61.1 (5.8)		61.2 (5.9)		0.920
**Family history of CRC**							
No	233	74.0%	85	65.9%	148	79.6%	**0.008**
Yes	33	10.5%	21	16.3%	12	6.5%	
Missing	49	15.6%	23	17.8%	26	14.0%	
**Diabetes**							
No	265	84.1%	116	89.9%	149	80.1%	**0.019**
Yes	50	15.9%	13	10.1%	37	19.9%	
**Hypertension**							
No	195	61.9%	86	66.7%	109	58.6%	0.147
Yes	120	38.1%	43	33.3%	77	41.4%	
**Depression**							
No	295	93.9%	120	93.8%	175	94.1%	0.902
Yes	19	6.1%	8	6.3%	11	5.9%	
**Tumour location**							
Distal	116	36.8%	57	44.2%	59	31.7%	**0.002**
Proximal	90	28.6%	23	17.8%	67	36.0%	
Rectal	109	34.6%	49	38.0%	60	32.3%	
**Other chronic diseases**							
No	82	26.0%	48	37.2%	34	18.3%	**<0.001**
Yes	233	74.0%	81	62.8%	152	81.7%	
**Cardiovascular event**							
No	293	93.0%	121	93.8%	172	92.5%	0.650
Yes	22	7.0%	8	6.2%	14	7.5%	
**Stroke/neurological event**							
No	309	98.1%	129	100.0%	180	96.8%	0.085
Yes	6	1.9%	0	0.0%	6	3.2%	
**Previous cancer diagnosis**							
No	269	85.4%	111	86.0%	158	84.9%	0.786
Yes	46	14.6%	18	14.0%	28	15.1%	
**Anticoagulants**							
No	271	86.0%	114	88.4%	157	84.4%	0.318
Yes	44	14.0%	15	11.6%	29	15.6%	
**Stage**							
0	27	8.6%	19	14.7%	8	4.3%	**<0.001**
I	70	22.2%	47	36.4%	23	12.4%	
II	66	21.0%	19	14.7%	47	25.3%	
III	103	32.7%	32	24.8%	71	38.2%	
IV	49	15.6%	12	9.3%	37	19.9%	

^a^ All variables except age are presented as number (%) of participants. Abbreviations: CRC, colorectal cancer; SD, standard deviation.

**Table 2:** Multivariable logistic model comparing symptom-based diagnoses with screening-based diagnoses.

	Odds Ratio	95% CI	*p*-Value
**Stage**			
0	1		
I	0.81	0.285 to 2.299	0.692
II	4.327	1.513 to 12.379	0.006
III	3.661	1.341 to 9.996	0.011
IV	5.732	1.862 to 17.645	0.002
**Family history of CRC**			
No	1		
Yes	0.291	0.124 to 0.679	0.004
Missing	0.645	0.313 to 1.329	0.234
**Diabetes**			
No	1		
Yes	2.308	1.059 to 5.032	0.035
**Tumour location**			
Distal	1		
Proximal	2.444	1.243 to 4.805	0.010
Rectal	0.842	0.458 to 1.548	0.580
**Other chronic diseases**			
No	1		
Yes	1.999	1.111 to 3.598	0.021

Total number of participants = 215; number of participants with symptom-based diagnosis = 186. LRT = 75 (*p* < 0.001); AUC = 0.774, 95% CI 0.723 to 0.825. Abbreviations: AUC, area under the receiver operating characteristic curve; CI, confidence interval; CRC, colorectal cancer; LRT, likelihood ratio test.

**Table 3:** Epidemiological analysis of prognosis at baseline (end of diagnostic testing; cross-sectional analysis) and at the end of follow-up.

	Global Prevalence	Prevalence in Symptoms-Based Diagnosis Patients (95% CI)	Prevalence in Screening-Based Diagnosis Patients (95% CI)	PR (95% CI)	AR (95% CI)	FAE
Stage IV (vs. 0-III)	0.16	0.20 (0.15 to 0.26)	0.09 (0.05 to 0.16)	2.11 (1.16 to 3.94) ^a^	0.11(0.02 to 0.18) ^b^	0.53
	Global incidence	Incidence in symptoms-based diagnosis patients (95% CI)	Incidence in screening-based diagnosis patients (95% CI)	RR (95% CI)	AR (95% CI)	FAE
CRC mortality	0.17	0.21 (0.15 to 0.27)	0.12 (0.06 to 0.17)	1.75 (1.04 to 3.13) ^a^	0.09 (0.01 to 0.17) ^b^	0.43
Other-cause mortality	0.07	0.08 (0.04 to 0.11)	0.06 (0.02 to 0.10)	1.33 (0.52 to 2.81)	0.02 (0.0 to 0.06)	0.25
All-cause mortality	0.24	0.28 (0.22 to 0.35)	0.18 (0.12 to 0.24)	1.60 (1.03 to 2.47) ^a^	0.10 (0.01 to 0.20) ^b^	0.38

^a^ *p* < 0.05 (95% CI does not include the null value of 1). ^b^ *p* < 0.05 (95% CI does not include the null value of 0). Abbreviations: AR, attributable risk; FAE, fraction attributable to exposure; PR, prevalence ratio; RR, relative risk.

**Table 4:** Cumulative incidence of total mortality by explanatory variables.

	Alive		Deceased		
Variable ^a^	n	%	n	%	*p* Value
**Type of diagnosis**					
Screening	106	82.2%	23	17.8%	**0.030**
Symptoms	133	71.5%	53	28.5%	
**Sex**					
Man	159	74.0%	56	26.0%	0.243
Woman	80	80.0%	20	20.0%	
**Age**					
Median (IQR)	61 (56–66)		64 (57–67)		**0.027**
Mean (SD)	60.7 (5.8)		62.4 (6.0)		
**Family background of CRC**					
No	180	77.3%	53	22.7%	0.504
Yes	25	75.8%	8	24.2%	
Missing	34	69.4%	15	30.6%	
**Diabetes**					
No	205	77.4%	60	22.6%	0.156
Yes	34	68.0%	16	32.0%	
**Hypertension**					
No	152	77.9%	43	22.1%	0.272
Yes	87	72.5%	33	27.5%	
**Depression**					
No	223	75.6%	72	24.4%	0.741
Yes	15	78.9%	4	21.1%	
**Tumour location**					
Distal	91	78.4%	25	21.6%	0.717
Proximal	67	74.4%	23	25.6%	
Rectal	81	74.3%	28	25.7%	
**Other chronic diseases**					
No	74	90.2%	8	9.8%	**<0.001**
Yes	165	70.8%	68	29.2%	
**Cardiovascular event**					
No	226	77.1%	67	22.9%	0.056
Yes	13	59.1%	9	40.9%	
**Stroke/neurological event**					
No	236	76.4%	73	23.6%	0.135
Yes	3	50.0%	3	50.0%	
**Previous cancer diagnosis**					
No	208	77.3%	61	22.7%	0.146
Yes	31	67.4%	15	32.6%	
**Anticoagulants**					
No	208	76.8%	63	23.2%	0.365
Yes	31	70.5%	13	29.5%	
**Stage**					
0	24	88.9%	3	11.1%	**<0.001**
I	64	91.4%	6	8.6%	
II	58	87.9%	8	12.1%	
III	77	74.8%	26	25.2%	
IV	16	32.7%	33	67.3%	

^a^ All variables except age are presented as number (%) of participants. Abbreviations: CRC, colorectal cancer; IQR, interquartile range; SD, standard deviation.

**Table 5:** Cox survival models for total mortality.

	Raw Adjustment			Model with Stage		
	HR	95% CI	*p*	HR	95% CI	*p*
**Type of diagnosis**						
Screening	1	-	-	NS		
Symptoms	1.66	1.02 to 2.72	0.041			
**Sex**						
Man	1	-	-	NS		
Woman	0.76	0.45 to 1.26	0.280			
**Family history of CRC**						
No	1	-	-	NS		
Yes	1.16	0.55 to 2.44	0.696			
Missing	1.42	0.80 to 2.51	0.236			
**Diabetes**						
No	1	-	-	NS		
Yes	1.50	0.85 to 2.57	0.164			
**Hypertension**						
No	1	-	-	NS		
Yes	1.30	0.81 to 2.01	0.287			
**Depression**						
No	1	-	-	NS		
Yes	0.87	0.32 to 2.39	0.793			
**Tumour location**						
Distal	1	-	-	NS		
Proximal	1.21	0.68 to 2.14	0.502			
Rectal	1.23	0.71 to 2.11	0.456			
**Other chronic diseases**						
No	1	-	-	NS		
Yes	3.24	1.55 to 6.74	0.002			
**Cardiovascular event**						
No	1	-	-	NS		
Yes	1.82	0.90 to 3.65	0.091			
**Stroke/neurological event**						
No	1	-	-	NS		
Yes	2.56	0.80 to 8.14	0.111			
**Previous cancer**						
No	1	-	-	1	-	-
Yes	1.47	0.83 to 2.58	0.183	2.57	1.40 to 4.72	0.002
**Anticoagulants**						
No	1	-	-	NS		
Yes	1.33	0.73 to 2.43	0.343			
**Age** (years)	1.04	1.00 to 1.08	0.040	NS		
**Stage**						
0	1	-	-	1	-	-
I	0.80	0.20 to 3.20	0.753	0.74	0.18 to 2.94	0.665
II	1.15	0.31 to 4.34	0.835	1.10	0.29 to 4.18	0.879
III	2.63	0.80 to 8.70	0.113	2.52	0.76 to 8.35	0.128
IV	10.48	3.20 to 34.33	<0.001	12.35	3.74 to 40.83	<0.001

Total number of participants = 315; number deceased = 76; LRT = 73.3 (*p* < 0.001); C-index = 0.760 (95% CI 0.707 to 0.813). Abbreviations: CI, confidence interval; CRC, colorectal cancer; HR, hazard ratio; LRT, likelihood ratio test; NS: not significant.

## Data Availability

The data that support the findings of this study are available from the first author S.A.N. or the corresponding author A.L.-P., upon reasonable request.
